# Plasma brain-derived neurotrophic factor levels, learning capacity and cognition in patients with first episode psychosis

**DOI:** 10.1186/1471-244X-13-27

**Published:** 2013-01-15

**Authors:** Sonia Ruiz de Azua, Carlos Matute, Laura Stertz, Fernando Mosquera, Aitor Palomino, Iris de la Rosa, Sara Barbeito, Patricia Vega, Flávio Kapczinski, Ana González-Pinto

**Affiliations:** 1 CIBERSAM (Biomedical Research Center in Mental Health Net), University Hospital of Alava, University of the Basque Country, 29 Olaguibel St, 01004, Vitoria, Spain; 2 CIBERNED. Neuroscience Department and Achucarro Basque Center for Neuroscience, University of the Basque 514 Country, 48940, Vizcaya, Spain; 3 Bipolar Disorders Program & INCT Translational Medicine, Hospital de Clínicas de Porto Alegre, Universidade Federal do Rio Grande do Sul, 2350 Ramiro Barcelos St, 90035-003, Porto Alegre, Brazil

**Keywords:** Psychotic disorder, Brain-derived neurotrophic factor, Schizophrenia, Cognition

## Abstract

**Background:**

Cognitive impairments are seen in first psychotic episode (FEP) patients. The neurobiological underpinnings that might underlie these changes remain unknown. The aim of this study is to investigate whether Brain Derived Neurotrophic Factor (BDNF) levels are associated with cognitive impairment in FEP patients compared with healthy controls.

**Methods:**

45 FEP patients and 45 healthy controls matched by age, gender and educational level were selected from the Basque Country area of Spain. Plasma BDNF levels were assessed in healthy controls and in patients. A battery of cognitive tests was applied to both groups, with the patients being assessed at 6 months after the acute episode and only in those with a clinical response to treatment.

**Results:**

Plasma BDNF levels were altered in patients compared with the control group. In FEP patients, we observed a positive association between BDNF levels at six months and five cognitive domains (learning ability, immediate and delayed memory, abstract thinking and processing speed) which persisted after controlling for medications prescribed, drug use, intelligence quotient (IQ) and negative symptoms. In the healthy control group, BDNF levels were not associated with cognitive test scores.

**Conclusion:**

Our results suggest that BDNF is associated with the cognitive impairment seen after a FEP. Further investigations of the role of this neurotrophin in the symptoms associated with psychosis onset are warranted.

## Background

Cognitive deficits have been described in patients with psychotic illnesses. More specifically, studies have found alterations in executive functioning, verbal memory and attention from the onset of psychosis [1,2]. Although the presence of these cognitive deficits is evident during the acute episode, it has also been established that patients diagnosed with chronic schizophrenia or bipolar disorder continue to have cognitive deficits [3].

Brain-Derived Neurotrophic Factor (BDNF) is a neurotrophin that has been associated with some mental diseases, such as depression [4], Alzheimer’s disease [5] and psychotic disorders [6,7]. BDNF is highly expressed in the cerebral cortex and hippocampus, brain areas known to regulate functions such as memory and emotion [6]. There is a correlation between hippocampal volumes and serum BDNF levels, such that smaller hippocampal volumes in drug-naïve patients with first episode psychosis are associated with lower BDNF levels [8]. A genetic interference of BDNF secretion could lead to deficits in cognition [5]. BDNF genetic polymorphisms have been associated with volume reductions in the central nervous system (CNS), such as smaller temporal and occipital lobar gray matter volumes [9,10] and abnormal hippocampal activation [11]. Previous research has found an association between BDNF Val66Met polymorphism and cognition: schizophrenic patients who are BDNF met carriers have a specific attention and visuospatial/constructional impairment [10,12], and BDNF met carriers with bipolar disorder have a specific alteration in executive function [13]. Preclinical investigations in animals indicate that BDNF promotes long-term potentiation [14]. Some studies in first-episode psychosis patients found that pharmacological treatment with olanzapine [15] or aripiprazole [16] is associated with increased BDNF levels. A recent clinical study has shown that bipolar patients who respond well to lithium treatment perform better in a battery of neuropsychological tests and have higher BDNF plasma levels than patients with a poor response to lithium and are similar to healthy controls [17]. A recent review of serum BDNF levels in patients with a first psychotic episode shows a significant decrease in patients with schizophrenia but not in those with non-schizophrenia psychosis [18]. It is thought that the measurement of peripheral markers, including BDNF, may increase our understanding of neuropsychiatric disorders [19-21].

To date, there is a lack of information about the relationship between BDNF levels and neurocognitive performance in patients with a first psychotic episode. Some animal studies [22] have suggested there could be a positive correlation between BDNF levels in the brain (especially the frontal cortex and hippocampus) and those in plasma, supporting the use of plasma BDNF levels as a biomarker of brain BDNF levels.

We investigated whether plasma BDNF levels are associated with cognitive impairment in patients with a first psychotic episode. We hypothesized *a priori* that plasma BDNF levels would be positively associated with cognitive functioning both in patients and in a healthy control group.

## Methods

### Participants

Between 2003 and 2005, 47 Caucasian patients with a first episode psychosis were recruited to the study while they were inpatients in the acute phase of illness and were followed up for 6 months. Participants were from the Alava catchment area in Spain and gave written informed consent to participate in protocols approved by the Basque Country Ethical Committee.

Inclusion criteria for patients were: age between 18 and 50 years at the time of first evaluation; and contact with mental health services for the first time with a diagnosis in the schizophrenia spectrum or a mood disorder with psychotic symptoms as defined by DSM-IV [23]. Patients were excluded if they had psychotic symptoms lasting longer than 6 months from inception to the time they were assessed for study eligibility. Other exclusion criteria were: presence of a concomitant Axis I disorder, with the exception of drug or alcohol abuse; mental retardation if functioning was impaired prior to onset of the disorder; pervasive developmental disorders; neurological diseases; history of head trauma with loss of consciousness; and pregnancy.

### Assessment

#### Clinical assessments

Psychiatric diagnoses were made by two clinical psychiatrists at baseline (i.e. upon hospitalisation for the acute episode) using the Structured Clinical Interview for DSM-IV-Axis I [24]. The diagnoses were subsequently reviewed and revised at the 6-month follow-up assessment and the 6-month diagnoses were used to describe the patients. Pharmacological treatment was prescribed according to the usual practice of the psychiatrist.

Patients were clinically assessed at baseline and at 6 months after the acute episode using the Positive and Negative Symptom Scale (PANSS) [25], the 21-item Hamilton Depression rating scale (HAMD-21) [26] and the Addiction Severity Index (ASI) [27] for use of drugs and alcohol. Drug and alcohol use was then assigned into one of 4 categories (no use, use, abuse, and dependence) following the criteria described in a previous study [28]. For describing the sociodemographic characteristics of participants (Table 1), the 4 categories were re-grouped into either use or no use.

**Table 1:** Sociodemographic characteristics of the first psychotic episode patient sample and healthy controls

	**Patients (*n*** **= 45)**	**Controls (*n*** **= 45)**	***χ***^**2**^	***P *value**
**Age**, median (SD)	24.3 (8.5)	24.0 (8.8)	−0.146	0.884
**Marital status**, *n* (%)				
Single	41 (91.1)	39 (86.7)	6.621	**0.036**
Married	1 (2.2)	6 (13.3)		
Divorced	3 (6.7)	0 (0)		
**Years of education**, *n* (%)				
Primary	5 (11.1)	4 (8.9)	3.401	0.334
Obligatory	18 (40)	11 (24.4)		
Secondary	16 (35.6)	24 (53.3)		
University	6 (13.3)	6 (13.3)		
**Employment**, *n* (%)				
Student	14 (31.1)	27 (60)	13.425	**0.004**
Unemployed	14 (31.1)	5 (11.1)		
Working	12 (26.7)	0 (0)		
Sick leave	5 (11.1)	13 (28.9)		
**Diagnosis**, *n* (%)				
Schizophrenia	25 (55.6)			
Affective disorder	12 (26.7)			
Non specific psychosis	8 (17.8)			
**Drug use,***n* (%) yes				
Tobacco	29 (64.4)	8 (17.8)	20.240	**<0.001**
Alcohol	30 (66.7)	16 (35.6)	8.715	**0.003**
Cannabis	22 (48.9)	3 (6.7)	19.994	**<0.001**
Other drugs	13 (28.9)	0 (0)	15.195	**<0.001**

Education: Primary (3–6 years), Obligatory (6–16 years), Secondary (16–18 years), University (18–23 years).

*SD* Standard deviation.

Cognitive assessments at 6 months were only made on patients who met the pre-established criteria for cognitive evaluation, which was clinical recovery from the acute episode. The clinical team decided whether patients had recovered from their psychotic symptoms and were ready for cognitive evaluation by evaluating symptom severity during patient interview and from the scores on the PANSS positive symptom subscale (less than 4 in any item) and HAMD-21 (less than 7 in the total score). Consequently, two patients were considered not to be clinically recovered at 6 months and, thus, were excluded from the study.

Forty-five Caucasian healthy controls selected from the same catchment area were matched by age, gender and educational level to the patient sample. Control subjects were not on medication, did not abuse alcohol, did not use drugs, had no personal history of major psychiatric disorders, and had no major psychiatric disorders in their first-degree relatives. The healthy controls were clinically assessed once using the Structured Clinical Interview for DSM-IV-Axis I [24].

#### Cognitive assessments

A battery of cognitive tests was administered to the healthy controls and to the patients at 6 months after the acute episode when they had responded to antipsychotic treatment. The cognitive domains assessed were: ***immediate and delayed memory and learning capacity*** using the Wechsler Memory Scale [29] (Logical Memory and Verbal Paired Associates); ***working memory and executive function*** using the Fluency Assessment Scale, Trail Making Test B [30], Letter-Number Sequencing and Digits backward (Wechsler Adult Intelligence Scale, WAIS-III) [31], the interference score from the Stroop Test [32] and using the Wisconsin Card Sorting Test (WCST) [33]; ***intelligence (abstract reasoning and crystallized intelligence)*** using Similarities and Vocabulary (WAIS-III) [31]; ***attention and processing speed*** using Digits (WAIS-III), Digit-Symbol Coding [31] words and colours score from the Stroop Test [32] and Trail Making Test A [30].

### Blood collection

Blood samples were withdrawn from all study participants at baseline (study entry) in the morning (at approximately 8:30 a.m. after fasting overnight) by venipuncture into vacuum tubes containing ethylene diamine tetraacetic acid (EDTA). Plasma was prepared by centrifugation at 300 x *g* for 10 min and the resulting supernatant was removed and then frozen at −80°C, as described previously [20]. Patient blood was also collected at 6 months after the acute episode on the same day as the cognitive assessments and plasma prepared as described above.

#### BDNF sandwich enzyme-linked ImmunoSorbent assay (ELISA)

Plasma BDNF levels were measured using a BDNF Sandwich ELISA Kit, according to the manufacturer’s instructions (Millipore, USA, Cat. No. CYT306). Briefly, diluted plasma samples (diluted 1:100) and serial dilutions of the BDNF standards (ranging from 7.8 to 500 pg/ml BDNF) were incubated for 24 hours in 96-well immunoassay plates pre-coated with mouse anti-human BDNF monoclonal antibody. The plates were then washed and a biotinylated mouse anti-human BDNF monoclonal antibody (diluted 1:1000 with diluent) was added to each well and incubated for 3 hours at room temperature. After washing, a streptavidin-enzyme conjugate (diluted 1:1000) was added and incubated at room temperature for 1 hour. After further washing, a substrate solution was added to the plates to initiate a reaction, which was stopped after 15 min by adding the stop solution (HCl). The amount of BDNF was determined immediately by measuring absorbance at 450 nm using a microplate reader. The standard curve demonstrated a direct relationship between optical density and BDNF concentration.

### Statistical analysis

Statistical analyses were performed using SPSS version 15.0. The sociodemographic characteristics of the sample were described using means ± standard deviations (SD) or frequencies, depending on the nature of the variable. Cognitive performance comparisons between patients and controls were analysed using student *t* tests because the variances were homogeneous. Bonferroni corrections were applied and the stated level of significance was *P* = 0.01 for memory and learning, *P* = 0.005 for executive function and working memory, *P* = 0.012 for processing speed and attention, and *P* = 0.025 for intelligence. We performed multivariate regression analyses with the cognition domains and BDNF levels, which controlled for the effects of the following variables: IQ, negative symptoms, use of typical/atypical antipsychotics, alcohol abuse, tobacco use/dependence and drugs use/abuse. We also controlled the association for the possible confounding effects of sex and age. Associations between cognitive performance, plasma BDNF levels and factors that could influence the results were assessed using simple linear regression analyses. Finally, any variables that were significant in these analyses were introduced as independent variables in the multivariate regression analyses, with the cognitive dimensions as dependent variables.

## Results

### Sociodemographic characteristics

Table 1 summarises the sociodemographic characteristics of the patient sample (*n* = 45) and healthy controls (*n* = 45). The two groups were similar, with a median age of 24 years, 55.6% of participants were male, and the majority were single and lived with their parents or guardians (77.3% patients vs. 60% controls). Also, there was no significant difference in the years of education between the patient and control groups (Table 1). The majority of patients (88.8%) were antipsychotic treatment-naive at baseline. Immediately upon being hospitalised, all except one patient received antipsychotics: 22.2% were prescribed a typical antipsychotic (the majority received haloperidol, dose range 10–20 mg/day), 60% were prescribed an atypical antipsychotic (the majority received risperidone, dose range 6–9 mg/day, or olanzapine, dose range 10–20 mg/day), and 15.5% were prescribed a combination of atypical antipsychotics and mood stabilizers. Antipsychotic prescription remained stable over the subsequent 6-month period, with only small changes in dose.

Tobacco was smoked by 64.4% of the patient group but only 17.8% of the control group. The frequency of alcohol use/abuse was higher in the patient group (66.7%) than in the control group (35.6%). Overall, 48.9% of patients used or abused cannabis while only 6.7% of the control subjects used/abused cannabis. In addition, 28.9% of the patient group reported sporadic use of other drugs, such as cocaine, “ecstasy” (methylenedioxymethamphetamine, MDMA) and amphetamines, whereas none of the healthy matched controls reported use of other drugs.

### Cognitive performance

Table 2 summarises the mean cognitive performance scores for patients at 6 months after the acute episode and for healthy controls in the following domains: memory and learning, executive functions, working memory, processing speed, attention and intelligence. In the majority of these domains, the cognitive performance of patients was significantly worse compared with the healthy controls. There was a memory deficit in patients at 6 months follow-up after their first psychotic episode as shown by mean scores of around 8 for the memory tests (except learning curve). The memory performance of the control group was higher than that of the patient group.

**Table 2:** Mean scores (SD) for the cognitive tests in patients at 6 months after acute episode and in healthy control subjects

**Cognitive task**	**Controls**	**Patients**	**Confidence**	t*	***P *value**
	**(*n*** **= 45)**	**(*n*** **= 45)**	**interval**		
**Memory and learning**					
Immediate Logical memory	10.50 (2.81)	8.05 (3.06)	0.622-0.896	3.635	0.001*
Learning Curve	11.39 (2.76)	10.66 (2.90)	0.772-1.072	1.134	0.260
Immediate Verbal Paired Associates	10.84 (3.80)	7.68 (3.22)	0.679-0.904	3.860	<0.001*
Delayed Logical memory	10.74 (3.45)	7.87 (3.48)	0.671-0.912	3.610	0.001*
Delayed Verbal Paired Associates	9.87 (2.04)	8.26 (2.43)	0.577-0.907	3.114	0.003*
**Executive function/Working memory**					
Trail Making B	62.33 (23.36)	95.27 (42.87)	1.016-1.053	−4.525	<0.001*
WAIS-III Letter-Number Span	11.22 (3.42)	8.02 (2.99)	0.616-0.856	4.695	<0.001*
WAIS-III Digits	10.38 (3.06)	8.89 (2.60)	0.710-0.968	2.488	0.015
WCST Number of errors	102.93 (16.51)	86.20 (22.64)	0.936-0.982	3.975	<0.001*
WCST Perseverative errors	102.93 (16.51)	89.50 (27.82)	0.959-0.994	2.745	0.007
WCST% Conceptual level R	102.88 (15.73)	86.14 (22.64)	0.938-0.984	3.683	<0.001*
Number of words FAS	14.16 (3.51)	12.44 (4.62)	0.810-1.002	1.988	0.05
Category fluency (animals)	22.69 (5.83)	18.16 (5.69)	0.795-0.945	3.729	<0.001*
Stroop interference	54.80 (8.09)	50.36 (8.05)	0.875-0.987	2.596	0.011
**Processing speed and attention**					
Trail Making A	35.00 (16.51)	47.29 (17.45)	1.016-1.079	−3.432	0.001*
WAIS-III Digit-Symbol Coding	10.24 (3.72)	7.52 (3.45)	0.698-0.932	3.282	0.002*
Stroop words	49.95 (7.59)	44.47 (9.49)	0.873-0.978	3.008	0.003*
Stroop colours	45.77 (6.67)	39.40 (8.62)	0.841-0.955	3.894	<0.001*
**Intelligence**					
WAIS-III Vocabulary	10.95 (2.19)	9.15 (3.02)	0.633-0.920	3.134	0.002*
WAIS-III Similarities	10.76 (2.36)	8.55 (2.48)	0.547-0.850	3.978	<0.001*

* Student’s *t*-test statistic.

Standard ranges: memory and learning: low ≤ 7, normal = 8-12, high ≥ 13; WAIS-III: low ≤ 7, normal = 8-12, high ≥ 13; Stroop: low ≤ 39, normal = 40-60, high ≥ 61; WCST (Wisconsin Card Sorting Test): deterioration < 106; no deterioration > 107; Trail Making A: low ≤ 39; normal ≥ 40; Trail Making B: low ≤ 91; normal ≥ 92; FAS (Fluency Assessment Scale): low ≤ 9; normal ≥ 10.

The executive function scores of the patient group were significantly different from the control group, with the exception of number of words and digits (Table 2). In addition, the scores for verbal fluency, digits and Stroop interference in the patient group were within the normal range and did not differ significantly from the control group (after Bonferroni correction). All scores for the patients’ processing speed and attention were significantly different from those in the healthy controls. Although the intelligence scale (vocabulary and similarities) scores were significantly different between patients and controls, all these scores were within the normal range of 8–12.

### Plasma BDNF levels and cognitive subtests

Baseline plasma BDNF levels (ng/ml) were significantly lower in the patient group compared with the control group (patients 6.09 ± 3.70; controls 9.19 ± 4.21; *t* = 3.25; *P* = 0.002). There was no significant difference in plasma BDNF levels between patients at 6 months (mean = 8.17, SD = 3.85) and controls (mean = 9.19, SD = 4.21; *t* = 1.07; *P* = 0.290).

We observed a significant positive association between plasma BDNF levels at 6 months after first hospitalisation and several of the cognitive domains (Table 3): abstract reasoning and processing speed (Wechsler Adult Intelligence Scale: Similarities, Digit-Symbol Coding), and learning capacity and delayed memory (Wechsler Memory Scale: Logical Memory Learning Curve, Verbal Paired Associates I Learning Curve and Verbal Paired Associates II). Moreover, there was also a significant positive correlation between the performance of the Vocabulary test (IQ) and the results in Similarities, Digit-Symbol Coding and Verbal Paired Associates II (Table 3). Additionally, negative symptoms measured by the PANSS had a significant negative correlation with Similarities, but was not significantly associated with any of the other cognitive subtests. Tobacco use in patients was not significantly associated with cognition, although there was a trend of association with Digit-Symbol Coding (*P* = 0.050) and Learning Curve of Logical Memory (*P* = 0.054).

**Table 3:** Simple linear regression analyses results of the association between cognition and plasma BDNF levels, pharmacological treatment, IQ, negative symptoms or drug use in the first episode patient group at 6 months after first hospitalisation

	**Similarities**		**Digit-symbol coding**		**Logical memory learning curve**		**Verbal paired associates I learning curve**		**Verbal paired associates II**	
	***r***^**2**^	***ß *(*P*)**	***r***^**2**^	***ß *(*P*)**	***r***^**2**^	***ß *(*P*)**	***r***^**2**^	***ß *(*P*)**	***r***^**2**^	***ß *(*P*)**
BDNF plasma levels	0.194	0.468	0.130	0.397	0.291	0.559	0.140	0.409	0.118	0.382
	**(0.006)**		**(0.024)**		**(0.001)**		**(0.018)**		**(0.028)**	
Pharmacological treatment	−0.027	0.025	−0.025	−0.056	0.011	0.193	−0.002	−0.157	0.002	−0.117
	(0.883)		(0.743)		(0.245)		(0.346)		(0.305)	
Wechsler Vocabulary (IQ)	0.432	0.669	0.262	0.531	−0.028	−0.015	0.007	0.183	0.224	0.495
	**(0.000)**		**(0.001)**		(0.928)		(0.270)		**(0.002)**	
Negative symptoms (PANSS N)	0.257	−0.530	0.081	−0.335	−0.033	−0.014	0.038	−0.264	0.052	−0.288
	**(0.002)**		(0.066)		(0.939)		(0.145)		(0.110)	
Tobacco use	0.034	0.246	0.080	0.325	0.074	0.315	−0.011	0.127	1.046	0.120
	(0.136)		(0.050)		(0.054)		(0.449)		(0.302)	
Alcohol use	−0.020	0.111	−0.020	−1.112	0.001	0.179	0.053	0.287	−0.017	0.120
	(0.538)		(0.541)		(0.320)		(0.105)		(0.505)	
Cannabis use	−0.021	−0.102	−0.017	−0.121	−0.027	0.067	0.001	0.178	−0.027	0.064
	(0.568)		(0.503)		(0.706)		(0.314)		(0.719)	
Other drugs use	0.025	0.083	0.059	−0.299	−0.018	0.117	−0.013	0.137	−0.032	−0.002
	(0.646)		(0.097)		(0.518)		(0.448)		(0.993)	

*r*^2^: Adjusted R-square; *ß*: Standardized correlation coefficient.

In the healthy control group, there was no significant association between plasma BDNF levels and any of the cognitive test results (Table 4). However, simple regression analyses found a significant positive correlation between Similarities performance and the Wechsler Vocabulary scale (IQ), Verbal Paired Associates II and tobacco use, and between Learning Curve of Logical Memory and age. In addition, the Digit-Symbol Coding results were predicted by the Vocabulary Scale (IQ), tobacco use and alcohol use (Table 4).

**Table 4:** Simple linear regression analysis results of the association between cognition and plasma BDNF levels, IQ, tobacco use and alcohol use in the healthy control group

	**Similarities**		**Digit-symbol coding**		**Logical memory learning curve**		**Verbal paired associates I learning curve**		**Verbal paired associates II**	
	***r***^**2**^	***ß *(*P*)**	***r***^**2**^	***ß *(*P*)**	***r***^**2**^	***ß *(*P*)**	***r***^**2**^	***ß *(*P*)**	***r***^**2**^	***ß *(*P*)**
BDNF plasma levels	−0.026	0.091	−0.011	−0.150	−0.034	−0.012	−0.034	0.010	0.035	−0.260
	(0.626)		(0.421)		(0.947)		(0.957)		(0.159)	
Wechsler Vocabulary (IQ)	0.332	0.591	0.137	0.401	−0.027	0.030	−0.014	−0.115	−0.026	0.044
	**(0.000)**		**(0.013)**		(0.859)		(0.493)		(0.791)	
Tobacco use	0.021	0.218	0.086	−0.332	−0.027	−0.027	−0.013	0.122	0.107	0.363
	(0.188)		**(0.042)**		(0.870)		(0.466)		**(0.025)**	
Alcohol use	−0.052	0.060	0.166	−0.458	−0.043	0.108	−0.042	0.114	−0.033	0.148
	(0.801)		**(0.042)**		(0.651)		(0.631)		(0.534)	

*r*^2^: Adjusted R-square; *ß*: Standardized correlation coefficient.

Finally, we performed one multivariate regression analysis for each of the five cognitive subtests (Similarities, Digit-Symbol Coding, Logical Memory, Verbal Paired Associates I, and Verbal Paired Associates II) including as independent variables those variables that were significant in the simple regression analyses. The Similarities variable model (*r*^2^ = 0.491) was predicted by the BDNF levels (*ß* = 0.349, *P* = 0.023) and IQ (*ß* = 0.663, *P* < 0.001), while negative symptoms measured by the PANSS negative scale were no longer significant. The performance of Digit-Symbol Coding (*r*^2^ = 0.328) was significantly associated with BDNF levels (*ß* = 0.325, *P* = 0.037) and IQ (*ß* = 0.467, *P* = 0.004). In the Learning Curve, Logical Memory (*r*^2^ = 0.291) and Verbal Paired Associates I (*r*^2^ = 0.123) were both significantly associated with BDNF levels (*ß* = 0.559, *P* = 0.001 and *ß* = 0.388, *P* = 0.025, respectively). Finally, delayed memory assessed using the Verbal Paired Associates II (*r*^2^ = 0.284) was significantly associated with BDNF levels (*ß* = 0.322, *P* = 0.041) and IQ (*ß* = 0.432, *P* = 0.008).

## Discussion

The results of the present clinical study show that plasma BDNF levels at 6 months after first hospitalisation for first episode psychosis (when patients have recovered from the acute episode) are positively associated with several cognitive domains. That is, cognitive performance is better in patients with higher BDNF levels. Specifically, we found positive associations between plasma BDNF levels and learning capacity (Logical Memory and Verbal Paired Associates I Learning Curve), verbal delayed memory (Verbal Paired Associates II), abstract verbal reasoning (Similarities) and processing speed (Digit-Symbol Coding) in patients. In contrast, an association between plasma BDNF levels and cognition was not seen in healthy control subjects. Similarly, some studies have found an association between immediate memory and BDNF levels in schizophrenic patients but not in healthy controls [34]. A possible explanation for our findings is that healthy volunteers and patients with higher BDNF levels may have sufficient cognitive reserve to compensate for other possible deficits present in patients with low BDNF levels; i.e., the brains of individuals with higher BDNF have a greater resistance to damage. Higher cognitive reserve has been shown to have a protective effect against dementia, schizophrenia and depression [35,36]. In the New Zealand Dunedin cohort, lower IQ was found to be a risk factor for the development of schizophrenia spectrum disorders [37]. In our sample, the healthy volunteer group had a higher IQ than the patient group and performed better in almost all of the cognitive tests. In both groups, IQ was positively associated with abstract thinking and processing speed.

Our results that patients with higher BDNF levels have better cognitive performance suggest that plasma BDNF levels could be used as a biological marker of cognition in patients with a first psychotic episode. Cognitive performance is an important clinical variable associated with prognosis in severe mental illness [3]. The nature of the relationship is unclear but, based on previous studies [38], we can hypothesize that lower BDNF functioning in the brain (e.g. during an acute episode of psychosis) can lead to cognitive impairment and could contribute towards the differences in cognition observed between different patients and between patients and healthy subjects. Other investigations have found positive relationships between BDNF levels and cognition in patients with diseases that result in cognitive impairment. One study found an association between maintained increases in serum BDNF levels and improved cognition in patients with schizophrenia, and suggested that serum BDNF levels could be used as a biological marker of cognitive improvement [38]. Another study found a positive correlation between serum BDNF levels and hippocampal volumes, which could explain the relationship between the BDNF and memory [8]. Moreover, BDNF levels could change during treatment with some medications. In patients with early Alzheimer’s disease, up-regulation of BDNF with lithium treatment has been associated with increases in cognitive performance [39]. In addition, some studies in rats have shown that BDNF mediates exercise-induced enhancements in learning and memory [40].

Plasma BDNF levels vary over time in psychosis [20] and schizophrenia [18], as demonstrated in the present study, where mean BDNF levels were lower during the acute first episode of psychosis and increased to normal levels (comparable to those in healthy subjects) after 6 months of naturalistic treatment when patients were in remission. Plasma BDNF levels have a significant negative correlation with positive symptoms at psychosis onset [41]. A recent study found that after 6 weeks of antipsychotic treatment, BDNF levels did not increase in patients treated with risperidone, haloperidol or olanzapine, but increased significantly in the subgroup treated with olanzapine [7]. Another study found a significant increase plasma BDNF levels during the first 6 months of follow-up after olanzapine treatment [15]. Recently, low serum levels of BDNF were found to be a state marker of depression that normalise during remission [42]. Our finding of a positive association between BDNF levels and cognition in FEP patients who have recovered from an acute episode of psychosis may be partly due to the effects of pharmacotherapy on BDNF levels in these patients.

In contrast to the variability of BDNF levels, cognitive impairments are more stable in patients with psychosis [43]. The observed associations between BDNF levels and cognition were significant when patients had recovered from their positive symptoms. These results raise the question of whether plasma BDNF levels may be used a possible marker beyond the acute clinical state.

The results of the multivariate regression models show that plasma BDNF levels are independently associated with several cognitive domains (learning capacity, verbal delayed memory, abstract verbal reasoning and processing speed); that is, patients with lower BDNF levels have a worse cognitive performance independent of premorbid IQ. However, our study shows that IQ is also an important independent factor associated with some cognitive domains (processing speed and delayed memory), as has been reported previously [44,45]. Cognitive performance has also been associated with negative symptoms [46]. We found that negative symptoms were inversely related to the Similarities subtest of the WAIS. However, the association was no longer significant when PANSS negative symptoms were introduced in the multivariate regression model together with BDNF and IQ.

This study has some limitations. The patients received pharmacological treatment in a naturalistic setting but we cannot analyse the possible differential effects of antipsychotics on BDNF because the sample is not large enough. In addition, other biological factors, such as oxidative stress [47] and environmental factors not measured in this study, could be associated with cognition. The small sample size can limit the statistical power. Nevertheless, the sample was large enough to suggest that BDNF and cognitive performance are associated, and that plasma BDNF levels after clinical stabilisation could be used as a biological marker of cognition in psychotic disorders.

## Conclusion

The cognitive performance of FEP patients is significantly lower than that of healthy control subjects across several cognitive domains. Plasma BDNF levels are associated with cognitive impairment in FEP patient but not in healthy controls. Cognitive performance is better in patients with higher BDNF levels when assessed after clinical stabilisation.

## Abbreviations

BDNF: Brain-derived neurotrophic factor; FEP: First episode psychoses; ELISA: Enzyme-linked immunosorbent assay; EDTA: Ethylene diamine tetraacetic acid; MDMA: Methylenedioxymethamphetamine; CNS: Central nervous system; IQ: Intelligence quotient; DSM-IV: Diagnostic and statistical manual of mental disorders; ASI: Addiction severity index; PANSS: Positive and negative syndrome scale; HAMD-21: Hamilton depression rating scale; WAIS-III: Wechsler adult intelligence scale; WCST: Wisconsin card sorting test.

## Competing interest

The authors have nothing to disclose financially and report no competing of interests.

## Authors’ contributions

LS, AP and CM were the basic scientifics and they did the biological analysis of the BDNF. SB and PV oversaw clinical management of the clinical and neuropsychological dates of the patients and controls. IdlR and FM were the clinicians who participate in the recruitment, diagnosis and the management of the pharmacological treatment. SRdA, LS, CM, FK and AG-P participated in the composition and in the edition of the manuscript, specially M, K and G-P professors were very useful assessors for the article. All authors read and approved the final manuscript.

## Pre-publication history

The pre-publication history for this paper can be accessed here:

http://www.biomedcentral.com/1471-244X/13/27/prepub
